# Extraction of a Large Central Airway Foreign Body Using Flexible Bronchoscopy Combined with an Endobronchial Blocker

**DOI:** 10.1155/2016/3179184

**Published:** 2016-05-04

**Authors:** Tyler Paradis, Michael Wollenberg, Brandon Tieu

**Affiliations:** ^1^Oregon Health and Science University, Portland, OR 97239, USA; ^2^Division of Cardiothoracic Surgery, Oregon Health and Science University, Portland, OR 97239, USA

## Abstract

Adult foreign body (FB) aspiration is an uncommon but potentially fatal event. Options for extraction include flexible bronchoscopy (FLXB), rigid bronchoscopy (RB), and surgical extraction. We report the case of a large, smooth aspirated rock causing airway obstruction in an elderly male. RB is generally the preferred approach for extraction of a large complex FB; however, due to its size, the FB had to be removed using FLXB combined with an endobronchial blocker. In this report, we describe the anesthetic and surgical considerations and the novel technique used to extract the FB.

## 1. Introduction

Foreign body (FB) aspiration uncommonly occurs in adult populations. Patients typically present with subtle, subacute symptoms with acute-on-chronic cough being the most common presenting symptom [1–3]. With adult FB aspiration being uncommon, standardized management is not well documented in the literature. Operative interventions include rigid bronchoscopy (RB), flexible bronchoscopy (FLXB), or retrieval via thoracotomy [3, 4]. FLXB is a reasonable option in cases of non-life-threatening FB aspiration. It is typically preferred for smaller objects in the lower airway, beyond the reach of rigid instruments. It is also useful in patients who are intubated and for those who have cervical instability or facial trauma [4–6]. Although not commonly used for this purpose in adults, FLXB has been found to be effective in recent studies. Sehgal et al. [7] reviewed over 25,998 flexible bronchoscopies in adults and found that 65 were done for FB retrieval, and when performed for this reason, their success rate was near 90%. Goyal et al. [3] reported similar success rates. We present a case of an adult patient who presented with respiratory distress and radiologic evidence of a large FB at the carina occluding 80% of the airway. The FB was retrieved using FLXB combined with an endobronchial blocker as a previously undescribed technique.

## 2. Case Report

**Figure 1:**
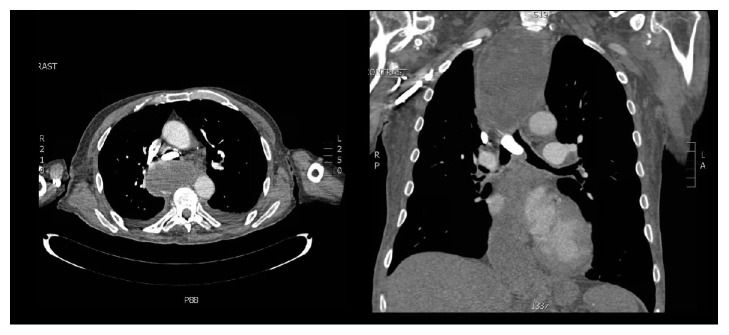
CT chest showing foreign body.

**Figure 2:**
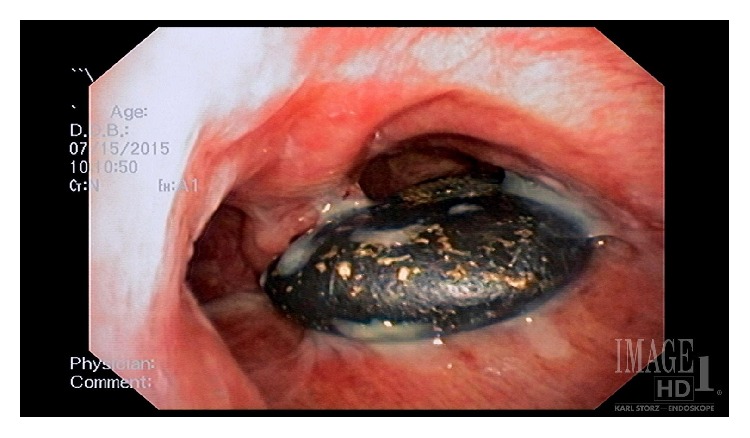
Bronchoscopy image showing the foreign body in the airway.

**Figure 3:**
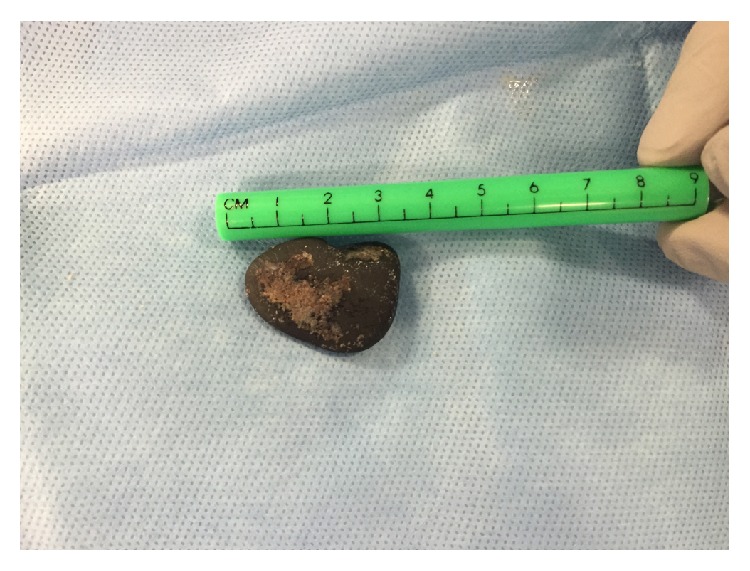
The foreign body after retrieval.

**Figure 4:**
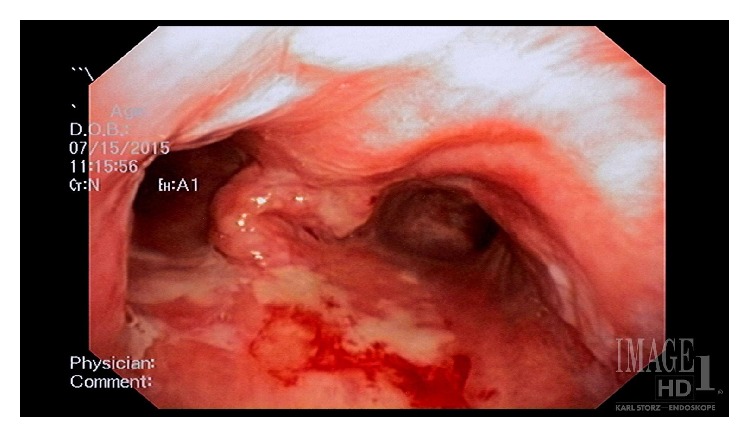
Postretrieval bronchoscopy image.

A 73 year-old male with a history of Chronic Obstructive Pulmonary Disease (COPD), hypertension (HTN), congestive heart failure (CHF) with reduced ejection fraction, paroxysmal atrial tachycardia, and dysphagia presented to a referring facility with hypoxia and was admitted for a presumed COPD exacerbation. The patient was placed on supplemental oxygen and noninvasive positive pressure ventilation with improvement in his blood oxygen saturation. Imaging showed an obstructing 2 × 3 cm FB at the carina (Figure 1) and a dilated esophagus. He was started on broad-spectrum antibiotics for aspiration pneumonia suspected on imaging. After an unsuccessful FLXB at the referring institution, the patient was transferred to our institution for further management. No additional description of the FB was available after the failed FLXB. The following day, the patient was taken to the operating room for retrieval of the FB. After preoxygenation, a rapid sequence induction with 100 mg of succinylcholine (PharMEDium, Lake Forest, IL, 60045, USA) and 120 mg of propofol (Fresenius Kabi USA LLC, Lake Zurich, IL, 60047, USA) was performed and the patient intubated with an 8.5 endotracheal tube (ETT). Anesthesia was maintained using a propofol infusion at 125 mcg/kg/minute. The patient was given 30 mg rocuronium (Hospira Inc., Lake Forest, IL, 60045, USA) for muscle relaxation and positive pressure ventilation was initiated. The patient remained on 100% oxygen throughout the procedure due to anticipated periods of apnea during extraction. Esophagoscopy was performed first, which showed a dilated esophagus filled with fluid and food debris. This was thought to be caused by a chronic esophageal dysmotility disorder but had not been formally evaluated at the time of presentation. We attempted to remove as much of the fluid and debris as possible prior to proceeding. Next, an adult flexible bronchoscope (Model BF 1TQ180, Olympus America, Center Valley, PA 18034, USA) was advanced through the ETT and a foreign body was identified at the carina (Figure 2). Up to this point, FLXB had been employed only to assess the characteristics of the FB and to formulate a plan for retrieval. The FB was identified as a landscaping stone measuring 2 × 3 cm (Figure 3). It did not appear to move with positive pressure ventilation. While the CT scan suggested 80% occlusion of the airway, on bronchoscopy we found the FB mainly obstructed the right mainstem bronchus only. The presence of granulation tissue beneath it suggested it had likely been present in the airway for an extended period of time. While we were prepared to perform a RB, it was determined that the RB accessories would not be able to retrieve the FB due the size, shape, and smooth surface of the FB. Prior to proceeding with thoracotomy, a decision was made to attempt retrieval of the FB with the flexible bronchoscope through an ETT. Several attempts were made at extracting the object including one attempt with alligator clamp, one with a basket, and one attempt using a Fogarty balloon® which was inflated distal to the mass. The stone was ultimately safely removed by placing a 9Fr endobronchial blocker (Model G44120, Cook Medical, Bloomington, IN, 47402, USA) via a pediatric bronchoscope (Model 11302BDD2, Storz Medical, Tuttlingen, 78532, Germany) passing through the ETT and gently directing the stone up and out of the patient's trachea. The endobronchial blocker was guided beyond the obstruction and inflated. The balloon inflated to a much larger size compared to the Fogarty balloon and was able to prevent the stone from backsliding. While positioning and gravity are sometimes used to assist extraction, we found the FB was able to be manipulated and extracted without changing the bed position. By holding ventilation and gently withdrawing the bronchoscope, endobronchial blocker, and ETT in a coordinated manner, the FB was brought to the vocal cords and the patient extubated at the same time. With gentle traction on the bronchial blocker and relaxation of the vocal cords with an additional 20 mg rocuronium, the FB came through the cords and was removed from the oropharynx with Magill forceps. The patient was reintubated without difficulty and the airway examined. The carina was noted to be edematous and friable but there was no defect in the airway (Figure 4). Underlying respiratory disease limited the time of total tracheal occlusion. The time from the onset of apnea to reintubation was approximately 30 seconds and no desaturation below 98% occurred. The patient made an uneventful recovery after FB extraction.

## 3. Discussion

An airway FB, which is large and anticipated to be difficult to remove, is typically removed using a rigid bronchoscope. Large foreign bodies in the central airway are high risk for completely occluding the airway, and a rigid bronchoscope allows for retrieval via direct access to the airway [3, 4]. Additionally, large foreign bodies typically require larger tools that can be passed through a rigid bronchoscope. Conversely, a flexible bronchoscope is typically used to retrieve smaller foreign bodies that have fallen distal to the major bronchi. While retrieval of a small FB with a flexible bronchoscope may take longer, the risk of complete occlusion of a major airway in this circumstance is usually low and with intubation and continuous positive pressure ventilation can be delivered during the retrieval process making the extraction less time sensitive. Smaller foreign bodies are also more easily retrieved with the limited tools that can be passed through a flexible bronchoscope [3–5]. Our patient presented with a large, heavy FB in the central airway. A FB of this size may present as a medical emergency due to risk of complete airway occlusion. Accordingly, a mass of this size would typically be retrieved using RB or require surgical intervention. This case is unique for several reasons. First, the patient was at high risk for aspiration; thus intubation for airway protection and retrieval of the FB via FLXB was desirable. Second, retrieval with the flexible bronchoscope through the ETT was advantageous as it allowed continuous positive pressure ventilation throughout the procedure, which avoided significant hypercapnia that can occur with jet ventilation routinely used with RB. This was of particular concern for our patient given his underlying COPD. Lastly, because we were able to ventilate and oxygenate around the FB, it afforded us the extra time to use FLXB to retrieve the FB. It is important to note, however, that because the bronchial blocker completely occluded the trachea, we were required to hold ventilation for a period of time making the actual extraction process particularly time sensitive. Due to the large size of the FB, we encountered the expected limitations of the flexible bronchoscope, namely, the lack of instruments to retrieve it. The instruments, which could be advanced through the flexible scope, were all designed to retrieve smaller objects. We remedied this problem by using the endobronchial blocker, which is mainly used to isolate one lung for pulmonary wedge resections and lobectomies as well as isolation of major, unilateral bronchopulmonary hemorrhage. We noted that the endobronchial blocker balloon inflated to a much larger size compared to the Fogarty balloon. Additionally, the endobronchial blocker offered the advantage of being placed next to the bronchoscope and not through it, thus offering an additional route for removing the FB.

This case is important because it identifies a technique not previously described where FLXB may be used to successfully retrieve large foreign bodies, which routinely require RB or surgical intervention for retrieval. This technique may be particularly important in those patients who cannot tolerate a RB such as those with neck instability or facial trauma [4, 6], those who may not tolerate the hypercapnia that can be associated with jet ventilation, or as in this case, those who require intubation. While retrieval was successful in this case, it is important to note that there is significant risk that with positive pressure ventilation in the setting of foreign body obstruction, a large central airway foreign body could become dislodged and completely obstruct the airway. Accordingly, intubation and positive pressure ventilation used for a FLXB should be used with caution in this patient population. While maintenance of spontaneous ventilation would usually be a reasonable solution, it may not be possible with this technique due to the patient coughing due to significant endobronchial manipulations. While not used in this case, one may also consider placing the bed in trendelenburg position, which can help prevent foreign bodies from advancement within the airway and potential occlusion. FLXB may be successfully employed in other similar cases of FB aspiration, thus avoiding RB or surgery. However, it hosts its own risks and should be considered as an alternative therapeutic intervention to the well-established techniques of RB and surgical extraction.
